# The Importance of Socioeconomic Status as a Modulator of the Bilingual Advantage in Cognitive Ability

**DOI:** 10.3389/fpsyg.2018.01818

**Published:** 2018-09-26

**Authors:** Kamila Naeem, Roberto Filippi, Eva Periche-Tomas, Andriani Papageorgiou, Peter Bright

**Affiliations:** ^1^Institute of Education, University College London, London, United Kingdom; ^2^Department of Psychology, Anglia Ruskin University, Cambridge, United Kingdom

**Keywords:** bilingual advantage, socioeconomic status, executive function, demographics, Simon task, Tower of London

## Abstract

Between-group variability in socioeconomic status (SES) has been identified as a potentially important contributory factor in studies reporting cognitive advantages in bilinguals over monolinguals (the so called “bilingual advantage”). The present study addresses the potential importance of this alternative explanatory variable in a study of low and high SES bilingual and monolingual performance on the Simon task and the Tower of London (TOL) task. Results indicated an overall bilingual response time advantage on the Simon task, despite equivalent error rates. Socioeconomic status was an important modulator in this effect, with evidence that bilingualism may be particularly important in promoting speed of processing advantages in low status individuals but have little impact in high status individuals. However, there was a *monolingual* advantage on the TOL test of executive planning ability. Together, our findings run counter to the central assertion of the bilingual advantage account, that the process of multi-language acquisition confers a broad cognitive advantage in executive function. We discuss these findings in the context of SES as an important modulator in published studies advocating a bilingual cognitive advantage.

## Introduction

According to recent estimates, more of the world’s population today is bilingual or multilingual than monolingual (Grosjean, 2010; Paradis et al., 2011). The dominant belief amongst academics until the 1960s was that second language learning had detrimental effects on cognitive development, particularly verbal IQ (e.g., Saer, 1923), and second language learning was discouraged (Hakuta and Diaz, 1985). This view was gradually overturned following the publication of a large scale study of middle-class monolingual and balanced-bilingual children attending French primary schools in Canada (Peal and Lambert, 1962). On the basis of their findings, these authors claimed that bilinguals typically show better mental flexibility, superior concept formation and higher IQ. In particular, their work indicated that bilingualism can confer general cognitive advantages which are not restricted to linguistic processing. Nevertheless, socioeconomic status (SES) was inadequately addressed as a possible alternative explanatory variable distinguishing the monolingual and bilingual groups, and the possibility that any bilingual advantage might be explained by such uncontrolled variables has become an important debate.

The likelihood that bilingual environments place disproportionately challenging demands on the developing brain is intuitively attractive and plausible if we accept the claim that cognitive resources must be allocated to the inhibition of one language while thinking or communicating in the other. The argument follows that these additional inhibitory demands underpin the development of our cognitive resources in such a way that would not typically be observed in monolingual contexts. Confirmatory evidence focused on and highlighted a bilingual advantage in inhibitory control (e.g., Bialystok, 1982, 2001), a position substantiated by a wealth of published evidence (e.g., Frye et al., 1995; Zelazo et al., 1996; Bialystok, 1999; Zelazo et al., 2003).

Much of the evidence for the bilingual advantage is based on performance on the Simon task, in which participants respond to the color of a stimulus, ignoring its position on the computer display. Typically, the stimulus is either green or blue and can be presented on the right or left of central fixation. In congruent trials the correct response (left or right) is aligned with its spatial position, but in incongruent trials the stimulus color/response mapping is crossed such that presentation on the left requires a right motor response and presentations on the right requires a left motor response. Reaction times are generally shorter for congruent trials than incongruent ones (known as the Simon effect), but this disparity is typically smaller for bilinguals than for monolinguals (Bialystok, 2006). Age has been found to influence the size of the bilingual advantage on this test, with evidence that the effect is particularly strong in older adults, indicating that lifelong experience of managing two languages may attenuate the age-related decline in inhibitory processing (Bialystok et al., 2004). Furthermore, the advantage was observed not only on the incongruent trials, suggesting that bilingualism may confer cognitive enhancement beyond inhibitory control *per se* and generalize to executive function more generally. These results have been replicated in subsequent research (Bialystok, 2006; Bialystok et al., 2006; Costa et al., 2008; Martin-Rhee and Bialystok, 2008), which has allowed refinement and clarification of the bilingual advantage to encompass monitoring (Costa et al., 2009), task switching (Prior and Gollan, 2011), and working memory (Luo et al., 2013; Kerrigan et al., 2017). Such work has encouraged reconceptualization of the bilingual advantage in terms of conflict monitoring (Costa et al., 2009) and general mental flexibility (Kroll and Bialystok, 2013). Synthesizing the findings from 31 studies, Hilchey and Klein (2011) concluded that, rather than reflecting advantages in inhibitory control, the bilingual advantage is better characterized as a domain-general “global advantage” in monitoring conflict and regulating task demands, and this explains the faster overall response times on both congruent and incongruent trials in conflict resolution tasks.

In the last decade, much of the research favoring the bilingual advantage has come under increasing scrutiny, with claims of poor experimental control, particularly with respect to matching of potential confounding variables across monolingual and bilingual groups. In particular, authors have claimed that alternative explanatory variables for intergroup differences, such as SES and other demographic or cultural factors, have not been systematically considered within study designs (for large-scale reviews see Paap et al., 2015; Lehtonen et al., 2018). Compounding this issue, evidence has been presented which indicates a lack of convergent validity across different tests developed to measure the same specific cognitive mechanisms thought to underpin the bilingual advantage (e.g., inhibitory control; Paap and Greenberg, 2013). Furthermore, another recent meta-analysis raises the complication that evidence for a bilingual advantage has been overplayed in the literature because of the tendency for journals to favor positive rather than null effects (i.e., publication bias; De Bruin et al., 2015b).

Whether the process of acquiring a second language confers a genuine cognitive advantage remains fiercely debated in the literature, and the counterclaim that factors independent of multi-language acquisition, such as SES, offer more plausible and parsimonious explanations for group differences in test performance is increasingly reported (e.g., Morton and Harper, 2007; Paap and Greenberg, 2013; Antón et al., 2014; Duñabeitia et al., 2014; Gathercole et al., 2014; Paap et al., 2015; Von Bastian et al., 2016; D’Souza et al., 2018; Goldsmith and Morton, 2018). In one of the earliest studies to question modern conceptualization of the bilingual advantage, Morton and Harper (2007) single out SES as a particularly important variable. They found a clear cognitive control advantage in children from high SES families relative to those from low SES families, but no evidence for performance differences between bilingual and monolingual children from the same socioeconomic backgrounds (see also Noble et al., 2005). Participants with low SES, particularly in young adult populations, are underrepresented in the literature on the bilingual advantage. However, one recent study has focused on non-linguistic executive control in Greek-Albanian young adult bilinguals from underprivileged social contexts, finding no bilingual advantage in interference control (Vivas et al., 2017).

In the present study we examine the effects of bilingualism and multilingualism on executive function in low and high SES, age-matched participants, addressing whether cognitive performance in young adults from underprivileged, low SES backgrounds might be disproportionately sensitive to factors associated with multilanguage acquisition. Given the interest of SES as an alternative explanatory variable for the bilingual cognitive advantage, we established this for each participant using stringent measurement criteria. The low SES bilingual group was composed of first-generation immigrants, half of whom had refugee and/or asylum seeker status. We employed two widely used tests in the literature on bilingual cognition and executive function, the Simon task and the Tower of London (TOL) task. To the extent that bilingualism, regardless of SES, confers an advantage in response inhibition, we predicted that bilinguals would perform disproportionately well on incongruent (conflict condition) relative to congruent (non-conflict condition) trials on the Simon task. Conversely, if bilingualism is associated with a more global cognitive monitoring advantage, they should perform proportionately better on both congruent and incongruent trials, relative to monolinguals. We also predicted that, if the bilingual advantage extends to planning and sustained cognitive control of behavior toward a goal, bilinguals should perform better on the TOL task.

## Materials and Methods

### Participants

The participants consisted of 90 adults aged between 18 and 30 years at the time of testing, of whom 45 were monolingual and 45 bilingual. Within each of these language groups, 20 had low SES and 25 high economic status, calculated on the basis of employment status and history, education and income. Age was statistically equivalent across language [*F*(1, 86) = 0.08, *p* = 0.76, eta-squared (η^2^)^1^ = 0.001] and SES [*F*(1, 86) = 0.19, *p* = 0.66, η^2^= 0.002] groups and the language by SES interaction effect was negligible [*F*(1, 86) = 0.039, *p* = 0.84, η^2^ = 0.000]. With respect to background cognitive performance, the language groups were equivalent on the Raven’s Matrices test of fluid intelligence [*F*(1, 86) = 0.095, *p* = 0.76, η^2^ = 0.001], digit span forward [*F*(1, 86) = 0.87, *p* = 0.35, η^2^ = 0.007] and backwards [*F*(1, 86) = 0.05, *p* = 0.82, η^2^ = 0.000] and although there was a highly significant main effect of SES (*p* < 0.001 in all cases), there were no significant language group by SES interaction effects (*p* = 0.76, η^2^ = 0.001; *p* = 0.96, η^2^ = 0.000; *p* = 0.16, η^2^= 0.019, respectively).

All low SES participants attended government-funded vocational courses at the same college in a predominantly low socioeconomic area in London, where they were recruited for participation in the current study. Although the monolingual controls were born and educated in the United Kingdom, the low SES bilinguals were immigrants of which half (*n* = 10) reported having refugee or asylum seeker status. The low SES participants were in receipt of financial social support, which was a condition for their participation and group allocation. High SES participants were recruited, using opportunity sampling, from local university and professional sectors in London.

Irrespective of SES (high/low), participants received their education in English, but the bilinguals spoke a language or languages other than English at home, and the majority reported using predominantly English to communicate outside of the home^2^. Among the low SES bilinguals, 18 claimed proficiency in a third language, 8 in a fourth language and 4 in a fifth language. Among the high SES bilinguals, 8 claimed proficiency in a third language and 1 in a fourth language. The monolinguals were not functionally proficient in any language other than English despite foreign language instruction in school.

All participants completed a language history questionnaire adapted from Li et al. (2006) and used in earlier studies by Filippi et al. (2012, 2015), which gathered language background and biographical information. The questionnaire items included details of employment, education and income to achieve a summary of SES. Additionally, bilingual participants provided language-related information, such as the number of languages acquired, years learning the second language, and individual self-rated competence in each language. Both objective information (e.g., years spent learning the second language) and subjective ratings on reading, writing, speaking and comprehension abilities indicated that all participants categorized as bilingual, irrespective of SES, were highly proficient in at least two languages (**Table 1**).

**Table 1:** Bilingual participants’ language history information.

	Low SES	*SD*	High SES	*SD*
Years learning L2	11.75	5.71	16.72	5.41
Self-rated L2 literacy	15.00	3.81	15.80	3.61
Self-rated L2 proficiency	14.50	3.75	17.40	2.38


*Sum of scores for L2 Reading and Writing was used to achieve self-rated L2 literacy, and Speaking and Comprehension to achieve self-rated L2 proficiency (each of the four skills were measured on a scale 1–10, where 1 = not literate and 10 = highly literate/proficient.*

This project was reviewed and approved by the UCL Institute of Education Research Ethics Committee. All participants gave informed consent prior to testing.

### Tasks

In addition to the tests of background cognitive ability (Raven’s Matrices, digit span forward and backwards), all participants were administered the Simon Test and the TOL task:

#### Simon Task

A computerized version of the Simon task (Simon and Wolf, 1963) was implemented in E-Prime (version 2.0; Schneider et al., 2002, 2007) and administered to all participants to assess inhibitory control based on stimulus-response conflict. The experiment was presented on a laptop computer with a 15.6-inch monitor and a two-button USB keypad connected to the laptop. Each trial began with a fixation cross (+) in the middle of the display that remained visible for 500 ms and was followed by a filled blue or red star (height = 1.7 cm, width = 1.8 cm on screen) displayed 3.9^o^ to the left or right of the fixation point. The goal was to press the corresponding key as quickly as possible according to the color of the star, which was presented for 1000 ms. The blue star was associated with the right index finger key on the keypad, whereas the red star was associated with the left index finger key. Blue and red dots were placed directly above the corresponding keys. Participants rested their index fingers on these keys and were instructed to press the key on the correct side according to the color of the stimulus, regardless of its position on the screen. Trials were defined as congruent if the color stimulus matched the key position (e.g., red star appearing on the left side of the screen required a left key response), and incongruent, when the color stimulus did not match the key position (e.g., red star appearing on the right side of the screen required a left key response). Participants scored one point when they pressed the correct key, with failure to respond within the 1000 ms stimulus presentation time classified as an error. There were in total 36 sequential randomized test trials, 18 congruent and 18 incongruent, with no practice trials. Raw scores were recorded as response times (RTs) and accuracy (proportion correct) for congruent and incongruent trial types.

#### Tower of London Task

A computerized version of the classic TOL task was administered to assess planning and problem solving (Berg and Byrd, 2002). In this version, the participants had to move colored discs on three pegs of different height to solve 12 problems of increasing difficulty in a fixed number of moves per trial (PEBL software, cf., Mueller and Piper, 2014). The computerized TOL instrument consisted of three piles of different height, the first of which could hold three discs, the second two discs, and the last only one disc. On each trial, the participant was shown a target disc configuration (top panel) and a start configuration (lower panel), each of which displayed three differently colored discs distributed across the three piles. The participant was required to move the discs in the lower panel to match the target configuration using the computer mouse. The number of possible moves was presented on a bar on the side of the screen, which reduced with each complete move. Twelve problems were presented, beginning with those that could be solved in two moves and progressing to those that required five moves. The trial was considered as successful if the solution was correctly submitted within the set number of moves. If the maximum number of moves was reached (irrespective of trial success) that trial terminated and the participant progressed onto the next problem. Scores were recorded as accuracy rates, the number of trials successfully solved, mean first-move latency, calculated as the length of time between the problem presentation and the first move, and mean total trial time.

### Design and Procedure

Participants were tested individually. The tasks were presented to all participants in a single session, which lasted between 40 min and 1 h including as many breaks between tasks as the participants wished to take. The order of the tests was as follows: Raven’s Progressive Matrices, digit span forward, digit span backward, Simon task, and TOL task. Raw data is provided online in **Supplementary Table 1**.

#### Materials

All tasks were presented on a laptop computer. Responses for the background measures were recorded by the experimenter on a scoring sheet. Simon and TOL data scores (response times and accuracy) were automatically recorded using E-Prime 2.0 software (Schneider et al., 2007) and stored electronically in a password-protected file. Additionally, the Simon task required the use of a Logitech Gamepad (model F310) and the TOL task was completed using an HP wireless computer mouse (model X3000) to ensure accuracy and ease of navigation.

## Results

### Simon Task Performance

We applied two three-way mixed ANOVA models, one on response times and one on accuracy, with congruency as a within-subjects variable (congruent/incongruent) and language group (monolingual/bilingual) and SES (low/high) as between-subjects variables. The analysis of response times identified a very robust main effect of congruency (i.e., a Simon effect), with longer response times on incongruent trials [*F*(1, 86) = 110.71, *p* < 0.001, η^2^= 0.563] but negligible congruency × language group [*F*(1, 86) = 0.06, *p* = 0.81, η^2^= 0.000], congruency × SES [*F*(1, 86) = 0.02, *p* = 0.88, η^2^= 0.000] and congruency × language group × SES [*F*(1, 86) = 0.02, *p* = 0.9, η^2^= 0.000] interaction effects.

**FIGURE 1:**
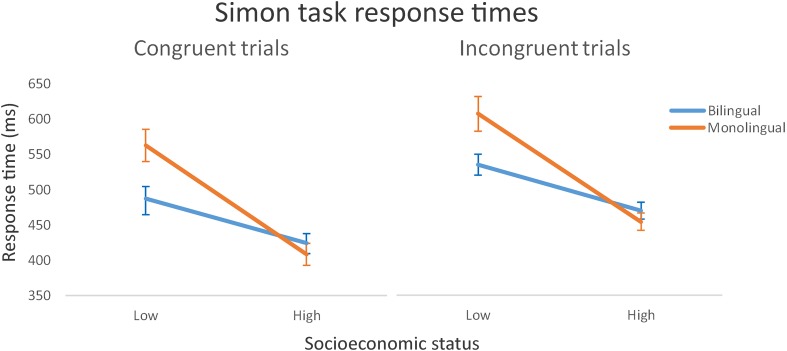
Mean Simon task response times (with standard error bars) for congruent and incongruent trials by group (bilinguals/monolinguals) and socioeconomic status (low/high).

There was a marginal main effect of language group [*F*(1, 86) = 3.236, *p* = 0.08, η^2^ = 0.022], with shorter response times in the BL group. The main effect of SES was, however, highly significant [*F*(1, 86) = 47.19, *p* < 0.001, η^2^ = 0.326], with high SES associated with shorter response times. The language group × SES interaction effect was also significant [*F*(1, 86) = 8.17, *p* = 0.005, η^2^ = 0.056]. The discrepancy in reaction times between low and high SES participants was disproportionately wider in monolinguals, indicating that the importance of SES in driving response times on the Simon task may be greater in monolinguals (**Figure 1**). Of particular interest here was the observation that although high SES MLs and BLs produced statistically equivalent response times [*F*(1, 48) = 0.87, *p* = 0.36, η^2^ = 0.018], low SES MLs produced statistically longer response times than low SES BLs [*F*(1, 38) = 7.22, *p* = 0.011, η^2^ = 0.160].

Mean accuracy performance was at/close to ceiling for congruent trials (0.966) but lower for incongruent trials (0.887). This effect of congruency was highly significant [*F*(1, 86) = 33.56, *p* < 0.001, η^2^= 0.278] but there were negligible congruency × language group [*F*(1, 86) = 0.02, *p* = 0.89, η^2^= 0.000], congruency × SES [*F*(1, 86) = 0.56, *p* = 0.46, η^2^= 0.005] and congruency × language group × SES [*F*(1, 86) = 0.49, *p* = 0.49, η^2^= 0.004] interaction effects. Accuracy performance was statistically equivalent across language groups [*F*(1, 86) = 0.55, *p* = 0.46, η^2^ = 0.006] and SES groups [*F*(1, 86) = 1.85, *p* = 0.178, η^2^= 0.021] and the language group × SES interaction effect was also non-significant [*F*(1, 86) = 1.11, *p* = 0.296, η^2^= 0.012].

### Tower of London Performance

**FIGURE 2:**
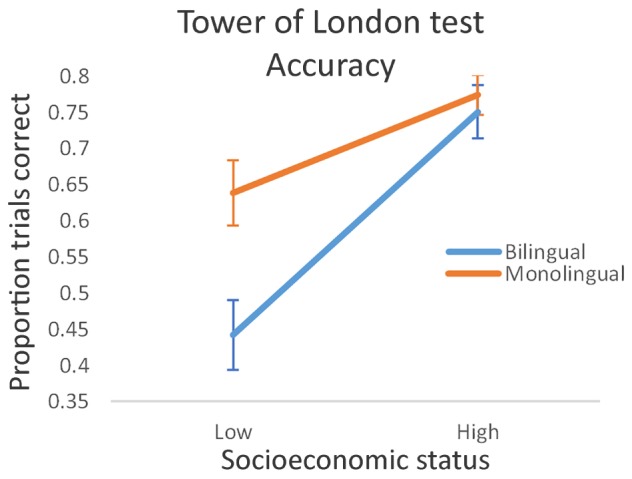
Tower of London test accuracy: proportion of trials correctly completed by low and high socioeconomic status bilinguals and monolinguals. Bars show standard error.

We applied two-way between groups analysis of variance models on accuracy, planning time and total response time. Each was specified with language group (monolingual/bilingual) and SES (high/low) as the between-subjects variables. There was a significant main effect of language group on accuracy (proportion of trials correct), with monolinguals outperforming bilinguals [*F*(1, 86) = 7.87, *p* = 0.006, η^2^ = 0.060]. There was also a highly significant main effect of SES, with high status conferring the accuracy advantage [*F*(1, 86) = 32.33, *p* < 0.001, η^2^ = 0.247]. The SES × language group interaction effect was also significant [*F*(1, 86) = 4.88, *p* = 0.030, η^2^ = 0.037]. The difference in performance between low and high SES participants was disproportionately large in bilinguals, with the low status bilingual participants failing to successfully complete more than half the trials on average (see **Figure 2**). Simple effects analysis confirmed the disproportionately poor performance in low SES bilinguals relative to low SES monolinguals [*F*(1, 38) = 8.79, *p* = 0.005, η^2^ = 0.188] and statistically equivalent high SES bilingual and monolingual performance [*F*(1, 48) = 0.26, *p* = 0.61, η^2^ = 0.006].

**FIGURE 3:**
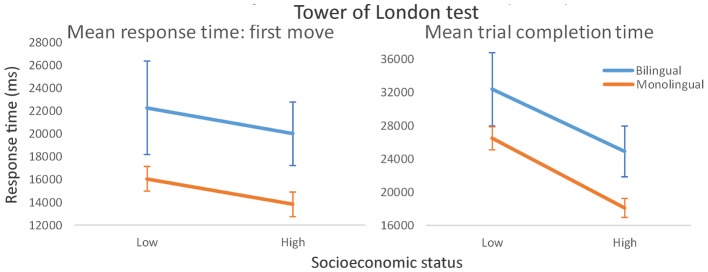
Tower of London mean response times: first move (an indication of planning time prior to trial execution) and trial completion time shown for low and high socioeconomic status bilinguals and monolinguals. Bars show standard error.

Time taken to produce the first move (an indication of solution planning time prior to execution) was compared across language and SES groups. Bilingual participants took significantly longer on this measure [*F*(1, 86) = 6.05, *p* = 0.016, η^2^ = 0.065], which when considered in the context of poorer overall accuracy, is clearly inconsistent with claims that bilingualism confers a broad intellectual advantage. The evidence against bilingual advantage theory is compounded by our observation that monolinguals also produced a shorter mean trial response time across the 12 trials [*F*(1, 86) = 5.32, *p* = 0.024, η^2^ = 0.053]. There was a main effect of SES on mean trial completion time, with faster timings produced by high SES participants [*F*(1, 86) = 8.31, *p* = 0.005, η^2^ = 0.083] but no main effect for first move response time [*F*(1, 86) = 0.80, *p* = 0.37, η^2^ = 0.009]. Language Group × SES interaction effects were non-significant in both cases (*p* > 0.8; **Figure 3**).

In summary, the bilingual advantage emerged in a marginal overall speed advantage in controlling interference (Simon task performance), but not in higher order cognitive processes involved in planning and problem-solving, as engaged by the TOL task. Socioeconomic status was identified as an important predictor of task performance in both tasks, and there was evidence from the Simon task that bilingualism may offset the response time disadvantage associated with low SES. Nevertheless, in our data, bilingualism conferred a performance disadvantage on the TOL test of planning and problem solving, and the accuracy disadvantage was particularly acute in those bilinguals with low SES, a finding incompatible with claims that multilanguage acquisition is associated with advantages in general mental flexibility and executive function. Although self-rated L2 proficiency was higher in high SES bilinguals [*t*(43) = 3.22, *p* = 0.002], correlations of proficiency with Simon and TOL task performance were negligible (*p* > 0.1 in all cases), and statistically controlling for this measure did not meaningfully alter the size of our reported effects. Correlational analyses conducted across the Simon and TOL tests revealed consistently small effect sizes, the largest of which (*r* = 0.271), between incongruent response times on the Simon task and total response time on the TOL test, revealed approximately 7% shared variance in these measures. Although correlations of performance on these tests with non-verbal general ability (as measured by Raven’s Matrices) were statistically equivalent across the monolingual and bilingual groups (*p* > 0.2 in all comparisons), these findings suggest that the Simon test and the TOL test may tap different mechanisms of cognitive control, and that these may be differentially influenced by the process of becoming bilingual.

## Discussion

The present study examined the effects of bilingualism on cognitive control and higher order executive function in low and high SES young adult bilinguals and monolinguals. We found a monolingual advantage in performance on the TOL task, which is not only incompatible with claims that bilingualism confers a general cognitive advantage in executive function, but infers that it may in fact obstruct the development of planning and goal-directed strategy formation. However, results from the Simon task indicate a marginal bilingual advantage in response times, irrespective of congruency (i.e., whether or not there was a strong demand on response inhibition), but that the advantage is modulated by SES. Our data raise the potentially important and intriguing possibility that multilanguage acquisition may be unimportant in high SES populations, but may help offset the negative impact that impoverished, low socioeconomic conditions have for the development of cognitive mechanisms underpinning information processing. In our study, low-status bilinguals showed significantly faster response times than low-status monolinguals, a pattern that was not replicated in high-status participants.

These findings help clarify the role of SES as a modulating influence on the likelihood that multilanguage acquisition will lead to cognitive advantages. The implication in the context of the size of our observed effects is that SES is the more important variable driving observed advantages, but that multilingual contexts may also be of significant benefit in environments in which access to economic, recreational and educational opportunities are relatively constrained. The advantage, however, appears to be quite specific. Low SES bilinguals performed disproportionately *poorly* on the TOL task (trials correctly completed) but high-status bilingual and monolingual participants’ performance was statistically equivalent. Bilinguals were also slower to produce their first move on this test (an indication of the need for longer planning time prior to execution) and to complete each trial. Therefore, a fractionation among components associated with cognitive control was apparent: bilinguals outperformed monolinguals on a task requiring monitoring and responding to compatible and incompatible stimulus-response mappings (the Simon task) and monolinguals outperformed bilinguals on a classic test requiring goal-directed strategic thinking and planning (the TOL test). While these observations cannot strictly be considered a double dissociation (all comparison groups are independent), we find it intriguing that well-matched groups undertaking the same tests under equivalent conditions have presented with a reversal of comparative performance as a function of our primary variable of interest (monolingualism/multilingualism). Our finding that SES influences the effect of multilanguage acquisition on performance in one of these tests but not the other, further complicates our ability to conceptualize the “bilingual advantage.”

How should we characterize, separate and distinguish between the cognitive mechanisms associated with the Simon and TOL tasks? Like the Simon task (and the Stroop test), the visual Flanker task incorporates the demand to suppress a prepotent/habitual response tendency (i.e., there is an incongruent stimulus/response mapping) which is compared with a non-conflicting/congruent response. Costa et al. (2009) employed versions of a flanker task which varied in their monitoring demands in young adult bilingual and monolingual university students and observed an overall bilingual speed advantage in the high-monitoring but not low-monitoring conditions, leading the authors to attribute the advantage to a more effective or efficient monitoring process (rather than, for example, an advantage in inhibitory control). Our data are also inconsistent with the inhibitory control explanation of the bilingual advantage, given that we observed virtually identical trends across monolinguals and bilinguals in both the congruent and incongruent Simon test conditions (a finding robustly supported in a large scale review by Hilchey and Klein, 2011). The model proposed by Costa et al. (2009) attributes the advantage to an enhanced cognitive flexibility for switching between contrasting demands associated with different task conditions (perhaps consistent with the way bilinguals disengage and engage between languages contingent upon social context). The authors further develop their theoretical framework by claiming that this monitoring advantage might incorporate an ongoing evaluation of the likely requirement for active attentional control (e.g., response suppression) given current task demands. That is, the real time processing advantage associated with bilingualism may occur before conflict resolution mechanisms are triggered.

The present findings are, in part, consistent with the kind of bilingual monitoring advantage described by Costa et al. (2009), but indicate that the capacity for bilingualism to confer such as an advantage is mitigated by situational conditions associated with SES. On the Simon task, only those with demonstrably low status benefitted from being bilingual, and the fact that, while the disparity in response times between low and high SES was smaller in bilinguals than monolinguals, high SES participants still responded numerically faster. It follows that SES appears to be a more important predictor of cognitive performance (as gauged by this task) than whether or not a person is bilingual. Nevertheless, implications for society of a significant beneficial cognitive impact in low socioeconomic populations are considerable, and we therefore recommend further studies employing a broader range of tasks and larger numbers of trials to examine the replicability of this finding and to further characterize the relationship.

The advantages observed on the Simon task did not transfer to TOL test performance: bilinguals were consistently slower in planning the moves required to match the target disk configuration and in executing those moves, and this was the case irrespective of SES. Compounding this evidence against any bilingual advantage in complex goal-relevant planning was the observation of disproportionately poor accuracy performance in low SES bilinguals. These findings are, in part, consistent with a study of simple and complex Simon task performance which indicated that bilingualism conferred advantages in selective attention specifically in the context of low working memory demand (Salvatierra and Rosselli, 2011). Other studies have reported equivalent monolingual and bilingual performance on the TOL test (e.g., De Bruin et al., 2015a; Cox et al., 2016) but, to our knowledge, the present study is the first to clearly indicate a disadvantage in a bilingual group. We suggest that the most likely reason for this disparity is that our study is also the first to explicitly recruit participants from the lowest level of SES (like the Cox et al., and de Bruin et al. studies, we observed similar performance in our other (i.e., high SES) monolingual and bilingual groups). Nevertheless, it is also possible that other experience-related factors operating in this group (half of whom were asylum seekers) underpinned the patterns of performance reported here, and more formal assessment of language fluency within and across comparison groups is encouraged. We also note recent evidence that, in young economically disadvantaged bilingual children with low proficiency in both languages, a stronger performance advantage over monolinguals was observed in tasks incorporating higher relative to lower cognitive control demands (Engel de Abreu et al., 2012).

We have recently reported evidence for a bilingual disadvantage in metacognitive processing (Folke et al., 2016), in which we employed a two-alternative-forced-choice task which required participants to determine which of two visually presented circles contained the most dots (with task difficulty systematically manipulated) and then state their confidence in their choice. We found that bilinguals were comparatively less confident on correctly completed trials and more confident on trials completed incorrectly. While purely speculative, one possible explanation for the patterns of TOL accuracy performance in the present study is that the cumulative effect of low SES and bilingualism might underpin comparatively low confidence in ongoing ability on this test, which, in turn, impacts on actual performance. In other words, if accurate monitoring of ongoing performance is not possible (i.e., on tasks in which our subjective assessment of our cognitive performance is poorly calibrated with objective performance) we cannot optimally regulate our knowledge or strategies in the service of goal attainment (see Bright et al., 2018, for further discussion of this theme). The TOL test is considerably more complex than the Simon test, incorporating strategic planning in order to determine moves that will bring the current disk configuration closer to the goal/target configuration, and subgoal conflicts, in which counterintuitive moves away from the goal state are sometimes required. This level of complexity, we would argue, renders performance on this test considerably more likely to be sensitive to the effects of poor metacognitive processing than is the case for the Simon test, which is operationally straightforward (i.e., restricted to processing binary congruent and incongruent stimulus/response mappings).

In summary, our findings are inconsistent with the claim that the process of acquiring a second language confers broad advantages in executive function. Instead, any cognitive advantage appears to relate to basic processing efficiency and is both contingent upon – and of secondary importance to – SES. Furthermore, this advantage may be offset by disadvantages in more complex tasks with stronger strategic and forward planning demands. We encourage further efforts toward isolating specific cognitive mechanisms that may be modulated positively or negatively through the process of multilanguage acquisition, and to carefully consider the moderating influence of situational, demographic and other factors.

## Author Contributions

KN collected the data and provided an early draft for this work, under the supervision of RF. RF and PB contributed equally to subsequent theoretical development, data analysis, and write-up for this paper. PB wrote the original submission and post-review revisions. EP-T and AP contributed to additional data collection and the manuscript editing. All authors read and approved the manuscript.

## Conflict of Interest Statement

The authors declare that the research was conducted in the absence of any commercial or financial relationships that could be construed as a potential conflict of interest.

## Footnotes

1 For all our reported ANOVA effects we manually calculated the eta-squared statistic (η^2^) as a measure of effect size rather than partial eta-squared, automatically provided in the SPSS statistical package. Apart from one-way ANOVAs (where the two values will be identical), η^2^ provides a more conservative estimate of the effect size.

2 The following second languages were represented in our bilingual group: Amharic, Arabic, Azeri, Bangla, Chinese, Darry, Farsi, French, Fur, Greek, Gujarati, Hindi, Hungarian, Irish, Italian, Koka, Kurdish, Lingala, Malay, Mauritian, Polish, Portuguese, Punjabi, Romanian, Russian, Spanish, Tagalog, Twi, Urdu, Zahau.
